# Research productivity across different ophthalmic subspecialties in the United States

**DOI:** 10.1186/s12913-019-4590-3

**Published:** 2019-11-01

**Authors:** Assaf Gershoni, Igor Vainer, Olga Reitblat, Francis B. Mimouni, Eitan Livny, Eytan Z. Blumenthal, Rita Ehrlich, Michael Mimouni

**Affiliations:** 1 Department of Ophthalmology, Rabin Medical Center – Beilinson Hospital, Petah Tikva, Israel; 2 Sackler Faculty of Medicine, Tel Aviv University, Tel Aviv, Israel; 3 Department of Ophthalmology, Rambam Health Care Campus, Haifa, Israel; 4 Department of Neonatology, Shaare Zedek Medical Center, Jerusalem, Israel

**Keywords:** Research productivity, H-index, Ophthalmic subspecialties, Academia

## Abstract

**Background:**

The purpose of this study was to compare the h-index, and subsequently the research productivity, among different ophthalmic subspecialties in the United States.

**Methods:**

A cohort of over 15,000 academic ophthalmologists residing in the United States (US) was identified out of the physician list of the American Academy of Ophthalmology. Of them, 1000 ophthalmologists with at least one publication were randomly retrieved, 100 in each of the following 10 subspecialties: cataract, cornea/external disease, glaucoma, medical retina, neuro-ophthalmology, pediatric ophthalmology, plastic/reconstructive ophthalmology, refractive surgery, retina/vitreous surgery and uveitis. Data collected included: number of published papers, h-index score, annual increase in h-index and the mean number of authors on each paper.

**Results:**

The mean h-index amongst all subspecialties was 9.87 ± 13.90, and the mean average annual increase in h-index was 0.22 ± 0.21. The mean number of papers published was 37.20 ± 80.08 and the mean number of authors on each paper was 3.39 ± 0.84. Uveitis was the most prolific subspecialty in mean number of papers (74.78 ± 131.37), in mean h-index (16.69 ± 20.00) and in mean annual increase in h-index (0.35 ± 0.28). The least fertile subspecialty with regards to research was cataract with 11.06 ± 27.65 mean number of papers, a mean h-index of 3.89 ± 5.84, and a mean annual increase in h-index of 0.11 ± 0.11.

**Conclusions:**

This study describes the research productivity in each ophthalmic subspecialty in the US, thus providing information on the research performance of each field and on the expected academic accomplishments within it.

## Background

The h-index was introduced in 2005 by prof. J.E. Hirsch to quantify the importance, significance and broad impact of a scientist’s cumulative research contribution [1]. The h-index reflects the number h of papers a researcher is a co-author on, each of which has been cited at least h times in other papers. Today it is widely accepted as the standard of scientometrics of an individuals’ research impact, and was further tailored to predict young scientists’ potential [2]. Furthermore, the h-index is used as an assessment tool in many universities and academic medical centers by committees for recruitment, promotion, tenure and awarding of grants. Its application in medicine has been recently explored in various specialties [3–12].

Ophthalmology encompasses a variety of activities which a physician can engage in: clinical medical care, surgical care, teaching and research. Ophthalmology residents interested in an academic career should be given the opportunity to get accurate information about the potential for research advancement and academic promotion of a given subspecialty they consider to fellow in. As there is a proven correlation between h-index, academic rank and grant funding [3, 4, 7, 11, 13–19], knowledge of the mean h-index in each field in ophthalmology and thus of the possibility of expected academic accomplishments might be factored in their decision-making process.

The purpose of this study was to describe and compare the research productivity (as measured by the proxy h-index) of various ophthalmic subspecialties in the United States (US). We hypothesized that authors from various sub-specialties of ophthalmology have different h-indices. It was also to try and determine the factors possibly associated with the h-index such as authors’ gender, total number of published papers, total number of citations, annual increase in h-index and number of authors on each paper. A last purpose was to determine the annual increase in h-index and the factors related to it.

## Methods

### Data acquisition

A study cohort of over 15,000 ophthalmologists residing in the US was identified out of the physician list of the American Academy of Ophthalmology. Each ophthalmologist was assigned a subspecialty, as recorded on the American Academy of Ophthalmology website (www.aao.org, accessed: December 4, 2018). Out of this cohort, 100 ophthalmologists were chosen in each one of the following 10 subspecialties: cataract, cornea/external disease, glaucoma, medical retina, neuro-ophthalmology, pediatric ophthalmology, plastic/reconstructive ophthalmology, refractive surgery, retina/vitreous surgery and uveitis. A random drown of ophthalmologists within each subspecialty, using random number allocating, was performed. In order to be chosen for the study, each ophthalmologist had to be one with at least one scientific publication. Ophthalmologists with no publications were excluded. Data collection was stopped once reaching a list of 100 ophthalmologists within each subspecialty. Overall, we retrieved 1000 ophthalmologists for this study.

Data were collected using Harzing, A.W. (2007) Publish or Perish (www.harzing.com, London. United Kingdom) on December 2018, and were incorporated into an Excel spreadsheet (Microsoft, Inc., Seattle, WA). The authors’ gender was obtained through a series of quests utilizing the Google search engine. The authors’ names were combined with their sub specialty, while relying on an official source (Hospital profile, Private website, Advertisement with a photograph, etc.), and their gender was recorded.

### Study variables

The primary outcome was to describe the research productivity by using the h-index, across the above-mentioned various ophthalmic subspecialties in the United States. Other variables which were collected included: total number of published papers, total number of citations, annual increase in h-index, number of authors on each paper and the gender of the ophthalmologists who were retrieved.

### Statistical analysis

Data were analyzed using the SPSS statistical software version 23.0 (SPSS, Cary, NC, US). Data are expressed as mean ± SD. Statistical differences between groups were tested using a one-way analysis of variance (ANOVA) for normally distributed data and the Kruskal-Wallis test for non-normally distributed data. The Bonferroni correction was applied for multiple comparisons. The Pearson correlation was used to analyze the relationship between h-index and the number of citations, the number of authors per paper and the number of papers. A *P*-value of less than 0.05 was considered statistically significant.

## Results

**Fig. 1:**
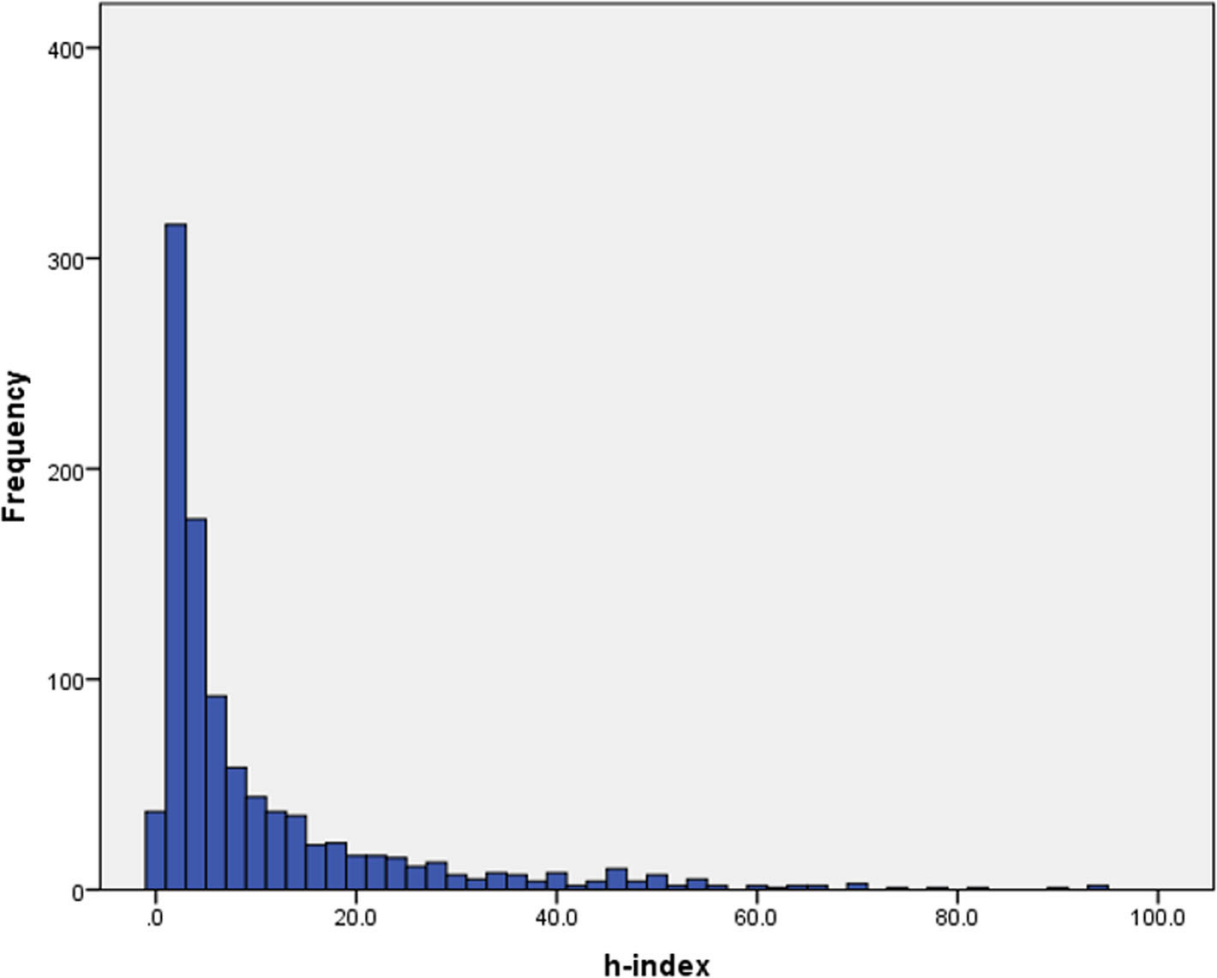
The h-index score histogram of the entire cohort

**Fig. 2:**
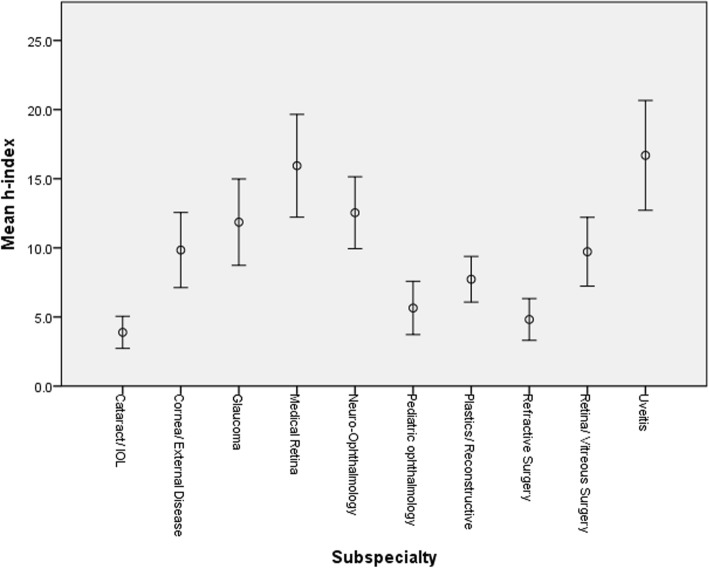
The h-index score in each of the 10 ophthalmology subspecialties

**Fig. 3:**
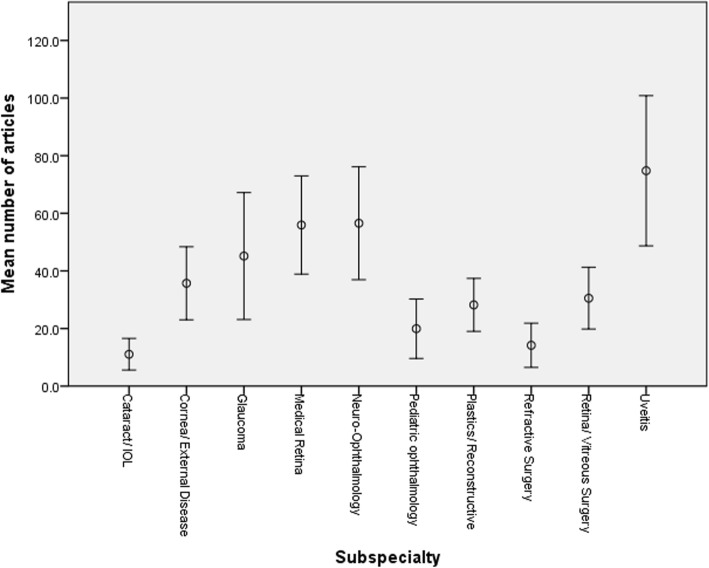
Number of articles for each of the 10 ophthalmology subspecialties

**Fig. 4:**
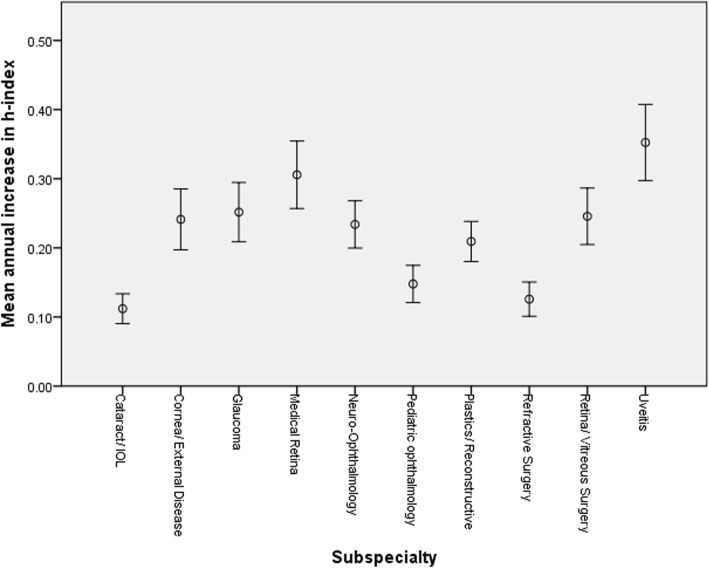
Average annual increase in the individual h-index score for each of the 10 ophthalmology subspecialties

Figure 1 depicts the frequency of the h-index score in the entire cohort. Figure 2 presents the mean h-index in each subspecialty. The mean h-index among all subspecialties was 9.87 ± 13.90 (Table 1). The mean number of papers published in the entire study group was 37.20 ± 80.08 and the mean number of authors on each paper was 3.39 ± 0.84 (Table 1). Figure 3 shows the mean number of papers an author published in each subspecialty. The mean average annual increase in h-index was 0.22 ± 0.21 (Table 1) and is shown for each subspecialty in Fig. 4.

**Table 1:** Research productivity comparison of ophthalmic subspecialties

Parameter	Num. of papers	Num. of authors for each paper	h-index	Average annual increase in h-index
Cataract/IOL	11.06 ± 27.65	3.33 ± 1.02	3.89 ± 5.84	0.11 ± 0.11
Cornea/External Disease	35.71 ± 63.90	3.56 ± 0.75	9.84 ± 13.70	0.24 ± 0.22
Glaucoma	45.15 ± 110.97	3.56 ± 0.68	11.86 ± 15.73	0.25 ± 0.22
Medical Retina	55.94 ± 85.93	3.52 ± 0.79	15.94 ± 18.74	0.31 ± 0.25
Neuro-Ophthalmology	56.55 ± 98.71	3.18 ± 0.75	12.54 ± 13.10	0.23 ± 0.17
Pediatric Ophthalmology	19.93 ± 51.98	3.41 ± 0.86	5.65 ± 9.70	0.15 ± 0.14
Plastics/Reconstructive	28.21 ± 46.34	3.08 ± 0.79	7.73 ± 8.32	0.21 ± 0.15
Refractive Surgery	14.17 ± 38.70	3.13 ± 1.07	4.82 ± 7.62	0.13 ± 0.13
Retina/Vitreous Surgery	30.53 ± 54.14	3.50 ± 0.75	9.72 ± 12.57	0.25 ± 0.21
Uveitis	74.78 ± 131.37	3.62 ± 0.71	16.69 ± 20.00	0.35 ± 0.28
Entire cohort	37.20 ± 80.08	3.39 ± 0.84	9.87 ± 13.90	0.22 ± 0.21

Values are mean ± SD

Uveitis was, the most prolific subspecialty in mean number of papers (74.78 ± 131.37, *P* < 0.001), in mean h-index (16.69 ± 20.00, *P* < 0.001) and in mean annual increase in h-index (0.35 ± 0.28, *P* < 0.001). The least fertile subspecialty with regards to research was cataract with a mean number of papers of 11.06 ± 27.65 and a mean h-index of 3.89 ± 5.84.

**Fig. 5:**
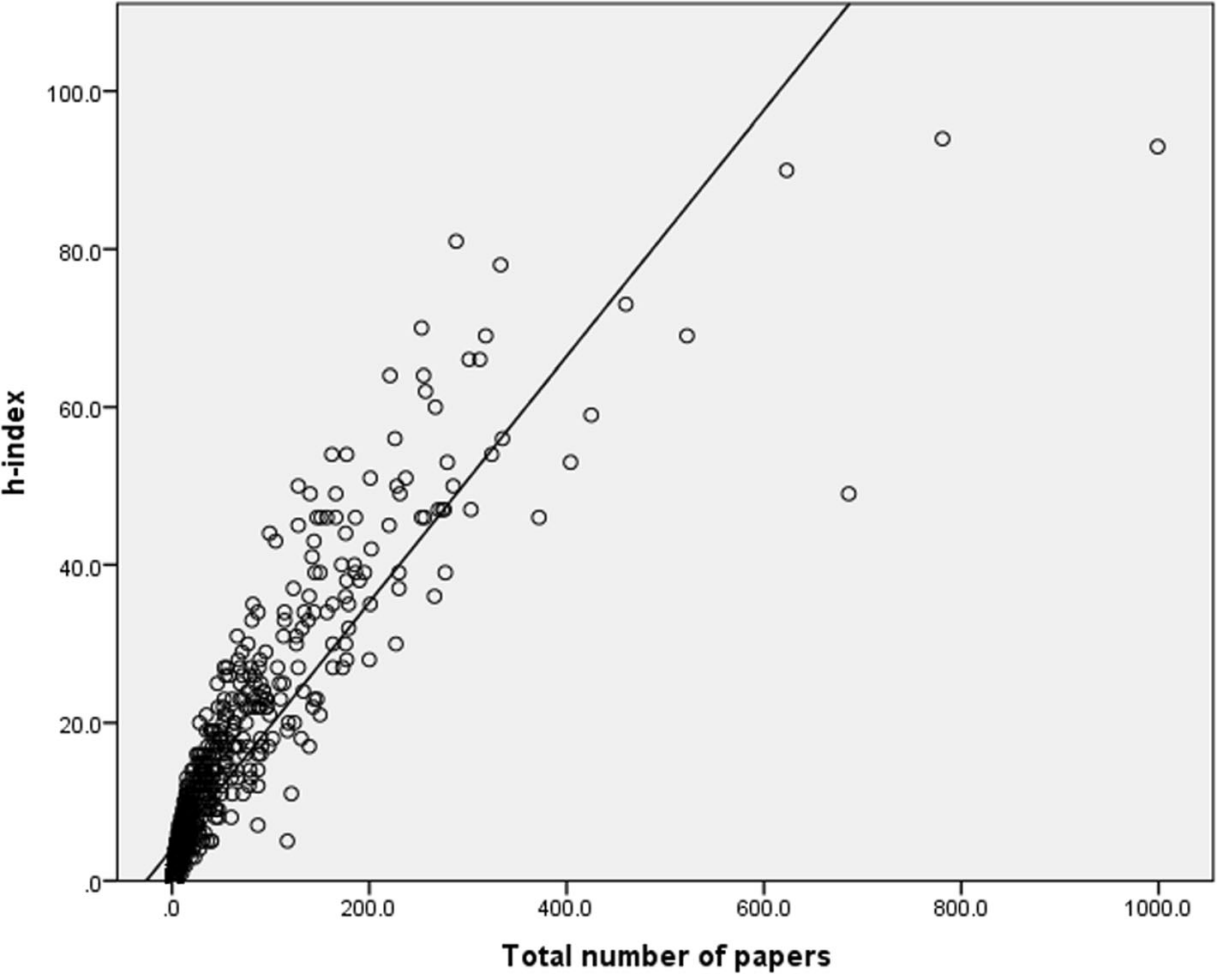
Correlation between the number of papers and the h-index

**Fig. 6:**
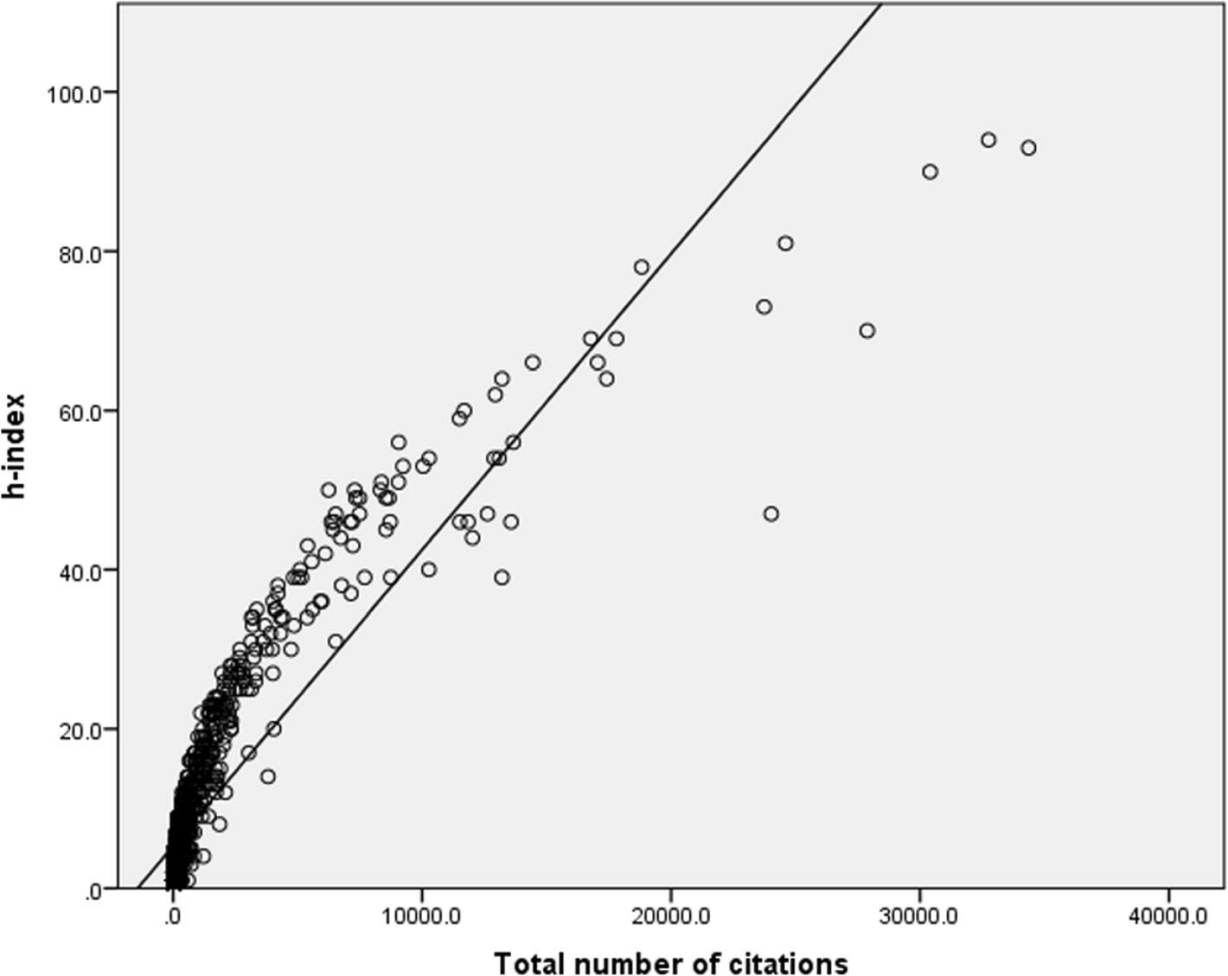
Correlation between the number of citations and the h-index

The mean number of authors on each paper ranged between 3.08 and 3.62 and was not found to be correlated with the h-index score (*r* = 0.083, *P* = 0.008). As expected, a significant high correlation was found between the number of papers published and the h-index score (*r* = 0.898, *P* < 0.001, Fig. 5), and between the number of citations and the h-index score (*r* = 0.893, *P* < 0.001, Fig. 6).

**Fig. 7:**
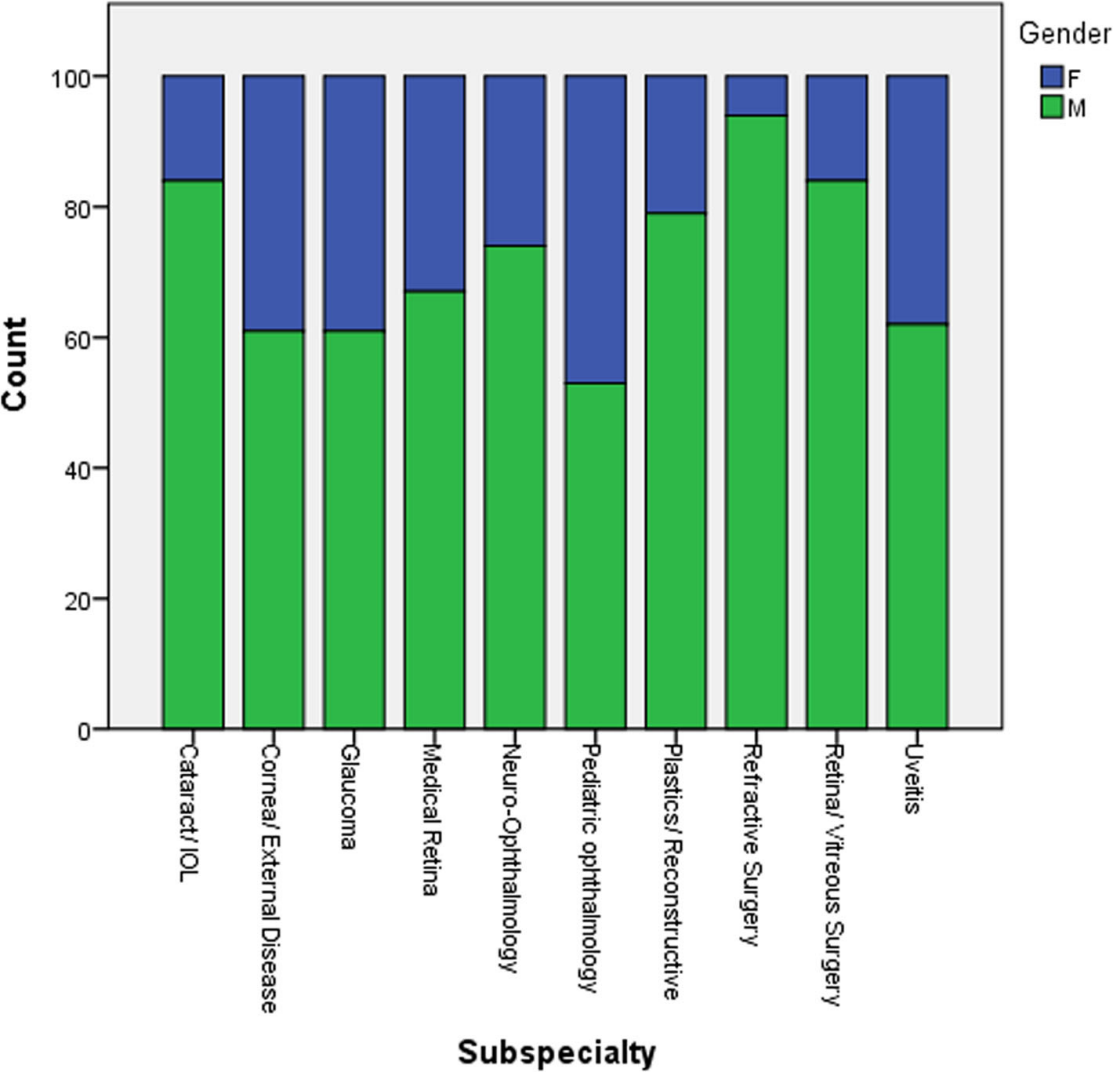
Distribution of genders in each of the 10 ophthalmology subspecialties

**Fig. 8:**
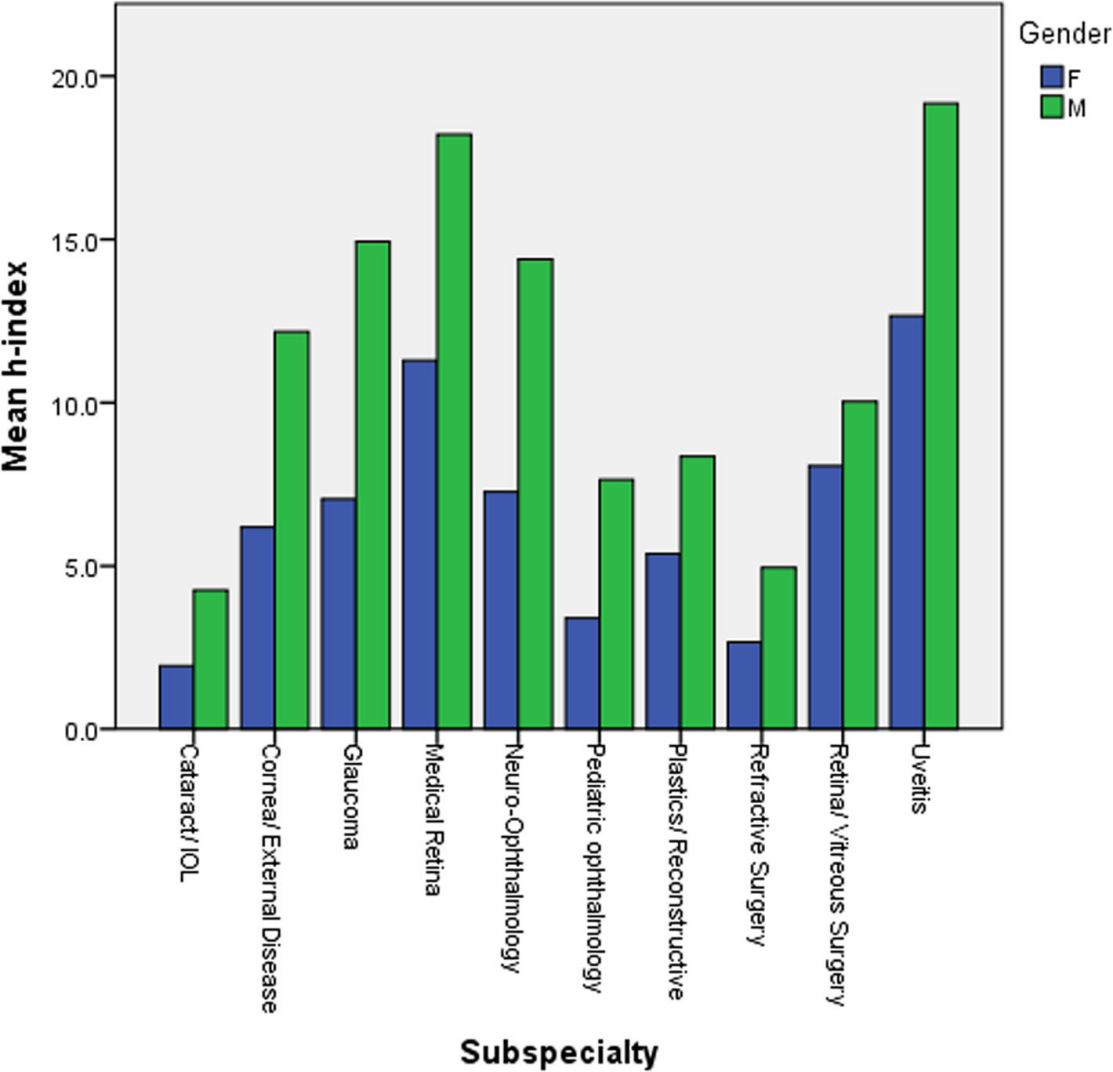
Comparison of the mean h-index between genders in each of the 10 ophthalmology subspecialties

The gender distribution of the ophthalmologists retrieved in each subspecialty is presented in Fig. 7. Figure 8 and Table 2 present a comparison of the mean h-index between genders in each individual ophthalmology subspecialty. In a comparison between genders in the entire cohort the male ophthalmologists exhibited a significantly higher mean h-index than the female ones (10.93 and 7.15, respectively, *P* < 0.001). There was a trend to higher mean h-index in males than in females in all subspecialties, which reached statistical significance in the fields of cornea/external disease, glaucoma, neuro-ophthalmology and pediatric ophthalmology (Table 2).

**Table 2:** Comparison of the mean h-index between genders in each individual ophthalmology subspecialty

Subspecialty	Males	Females	*P*
Entire cohort	10.93 ± 14.78	7.15 ± 10.94	< 0.001
Cataract/IOL	4.26 ± 6.28	1.94 ± 1.18	0.145
Cornea/External Disease	12.18 ± 16.21	6.18 ± 7.14	0.032
Glaucoma	14.93 ± 18.69	7.05 ± 7.33	0.014
Medical Retina	18.22 ± 19.15	11.30 ± 17.23	0.082
Neuro-Ophthalmology	14.39 ± 13.59	7.27 ± 9.99	0.016
Pediatric Ophthalmology	7.64 ± 12.49	3.40 ± 4.09	0.029
Plastics/Reconstructive	8.35 ± 8.74	5.38 ± 6.14	0.147
Refractive Surgery	4.96 ± 7.83	2.67 ± 1.97	0.478
Retina/Vitreous Surgery	10.04 ± 13.12	8.06 ± 9.28	0.568
Uveitis	19.16 ± 21.04	12.66 ± 17.71	0.115

Values are mean ± SD

## Discussion

The purpose of this study was to compare the h-index (a proxy measurement of research productivity) across various ophthalmology subspecialties in the United States. To the best of our knowledge, no such study has been conducted to date.

In 2005, prof. Jorge Hirsch introduced the h-index as an alternative to other scientific performance indices, such as number of publications, average number of citations and sum of all citations, in order to quantify the importance, significance and broad impact of a scientist’s cumulative research contributions [1]. The h-index reflects the number h of papers a researcher is a co-author on with at least h citations in other articles. It is widely accepted throughout the scientific community because it quantifies both the author’s scientific productivity and the cumulative impact of his work. Furthermore, Poynard et al. demonstrated that a high h-index was associated with true conclusions, methodological quality of trials and positive predictive values [20]. It has even been shown that the h-index, as a measure of scientific interest, appears to be a reflection of the true impact of human diseases in medicine [21]. In addition, not only is the h-index a score reflecting one individual’s lifetime work, but it has been further implemented to identify the research strength of institutions and to measure their academic productivity [22].

However, the h-index has its downsides as it can be inflated with self-citations [23] and may be discriminatory of epistemological beliefs and methodological preferences [24]. It can also act as a stressor and a source for burnout symptoms to young researchers who have yet to build their own, high h-index. In contrast, a high h-index may be reassuring for scientists and may become an important motivational factor for further, high quality research [25].

In this study, we compared the research productivity in 10 subspecialties of ophthalmology: cataract, cornea/external disease, glaucoma, medical retina, neuro-ophthalmology, pediatric ophthalmology, plastic/reconstructive ophthalmology, refractive surgery, retina/vitreous surgery and uveitis. The h-index score as well as the number of published papers were highest in uveitis followed by neuro-ophthalmology and medical retina. We speculate that, since these three fields are not surgical, they may grant more time to immerse in the minutiae of research. Theoretically, since these three fields complement other fields in medicine such as internal medicine and rheumatology with regards to uveitis, internal medicine with regards to medical retina and neurology and neurosurgery with regards to neuro-ophthalmology, they also might open the way to more collaboration with other practitioners, and hence more research opportunities. Against the latter theory is the lack of correlation that we found between mean number of authors and h-index. The mean h-index in uveitis was 16.69 and by comparison, in cataract the mean h-index score was 3.89 which was the lowest of all subspecialties. The other two subspecialties besides cataract with the lowest h-index, average annual increase in h-index and number of papers were pediatric ophthalmology and refractive surgery. Importantly, two of these subspecialties are essentially surgical ones, and possibly the most rewarding financially. Thus we speculate that such a financial incentive might harm academic motivation and performance. Regarding the annual increase in h-index, uveitis still ranked first with a mean increase of 0.35 to the h-index score annually and medical retina ranks second with 0.31.

Nevertheless, in terms of ophthalmology, this superiority in publication indices does not necessarily reflect medical and social impact, but only the scope of academic activity. This can be influenced by other factors, such as spare time for research, economic motives and other academic and medical disciplines. For example, as mentioned above, uveitis, medical retina and neuro-ophthalmology are all related to other medical specialties. Thus, the potential for publication and citation in these fields is beyond the bounds of ophthalmology journals; therefore, expanding academic productivity, but not representing their clinical centrality in ophthalmology.

As expected, a significant correlation was found between the number of papers and the h-index score and between the number of citations and the h-index score.

Another interesting aspect which was looked at was the gender distribution in each subspecialty. In a recent publication [26], we showed that despite an overall increase in the contribution of women to the field of ophthalmology, contributions to articles published in subspecialty ophthalmology journals and the proportion of women listed as last authors on overall articles published in ophthalmology journals are still low. In this study, we also found that that in all subspecialties there was a male predominance. In addition, the mean h-index of the male authors was significantly higher than that of the female ones in the entire cohort (Table 2), as well as in every subspecialty (Table 2, Fig. 8). Nevertheless, the recent increase in the contribution of women to the field of ophthalmology will hopefully begin to show its markings in the near future.

This study has several limitations. First, individuals in each field were chosen randomly, and even though the sample size was quite large, the samples may not be, by chance, representative of the group they were extracted from. Second, the sample included only ophthalmologists that published at least one paper. Including clinicians who have not published any papers would have lowered the study measures regarding cumulative academic contribution (mean number of published papers, mean h-index score and mean annual increase in h-index). However, the decrease in each subspecialty might have been different, and thus, possibly modifying the outcomes.

Notwithstanding its limitations, this study is the first to compare the research productivity among major subspecialties of opthalmology. It may provide young scientist in search of a fertile field of academic ophthalmology an additional tool for evaluation.

## Conclusions

In this study, we described the research productivity in different ophthalmic subspecialties in the US, providing, to the best of our knowledge for the first time, valuable information on the research performance of each field and on the expected academic accomplishments within it. As evident by the average h-index score and number of publications, we have found the highest academic productivity to be in uveitis followed by neuro-ophthalmology and medical retina.

## Data Availability

The datasets used and/or analysed during the current study are available from the corresponding author on reasonable request.

## Abbreviation

US: United States
